# Substance Use Among American Indian Youths Today: A Threat to Our Future

**DOI:** 10.1001/jamanetworkopen.2018.0381

**Published:** 2018-05-18

**Authors:** Spero M. Manson

**Affiliations:** Centers for American Indian and Alaska Native Health, Colorado School of Public Health, University of Colorado, Aurora

The worldview of American Indians turns on the Seventh Generation Principle, namely that decisions—personal, corporate, governmental—by those who came before us affect today and that the decisions we make now in turn affect those who follow, stretching 7 generations in each direction. This is the basis for the often-heard phrase in everyday discourse among Native people that “our youth are our future” and why we place such special store in youths’ health and well-being. They embody the continuity of the past, present, and future of our ways of life. As our children fare, so too we all fare. But mounting evidence chronicles the increasing peril of substance use among American Indian youths and fuels growing concern for the future of our youths and thus communities. The article by Swaim and Stanley^1^ is the most recent account to document the nature and extent of this condition among young Native people and the unsettling disparities between them and their counterparts in the general population. It is not surprising that the title of their survey is Our Youth, Our Future (OYOF).

Swaim and Stanley describe the results of their most recent phase of a long-standing, school-based epidemiologic study of substance use by American Indian youths living on or near reservations from across a geographically diverse swath of the country.^2^ Their current effort is distinguished by a pointed attempt to maximize comparison with similar questions asked by Monitoring the Future, a study of the behaviors, attitudes, and values of large samples of 8th-, 10th-, and 12th-grade students across the nation.^3^ Their key findings, cast in terms of self-reported lifetime and last-30-day use of alcohol, marijuana, and other drugs, are consistent with prior but much more select and partial studies of the nature of substance use among American Indian adolescents.^3^ Just as important, they permit direct comparison with the nation’s youths in general.

Focusing on participants in the eighth (n = 570), 10th (n = 582), and 12th (n = 508) grades, the OYOF survey revealed that American Indian students, compared with their Monitoring the Future counterparts, reported significantly higher use of virtually all substances except amphetamines and tranquilizers. These differences are greatest at eighth grade and continue, albeit slightly attenuated, into the 12th grade. Of special concern is that such disparities appear to be growing at an alarming rate. Swaim and Stanley note that, in reference to their previous 2014 survey, the relative risk of American Indian students using alcohol and marijuana in 2016–2017 did not change. However, the likely use of other drugs by Native youths increased substantially between these 2 periods.

There is little doubt about the value of continuing surveillance efforts such as the OYOF survey, especially given the changing character of substance use with respect to possible determinants, initiation, drug type, modes of delivery, perceptions of harm, and impacts of social policies. In this regard, the OYOF survey is a remarkable contribution to our ability to monitor and describe the landscape of substance use among American Indian adolescents. It is particularly valuable given the underrepresentation of Native youths in national epidemiologic studies.

While Swaim and Stanley carefully consider several important limitations to their findings, other limitations warrant additional attention. For example, previous studies describe marked tribal and geographic variation in substance use among American Indian students.^4^ The 31 schools participating in the OYOF survey are distributed across 7 regions, with nearly two-thirds of the students recruited from the Northern Plains (20.5%) and Southwest (43.3%). I wonder whether the analyses undertaken—which focus on the aggregate—may have obscured important regional differences among OYOF respondents. Moreover, these students attended schools on or adjacent to reservations, which are largely rural and probably tribal as well as public. Yet more than 70% of the American Indian population now lives in our nation’s cities.^5^ Although estimates of substance use among urban Native youths are far less well established, there is reason to suspect they may differ from those among Native youths who live in rural reservation communities, which introduces a cautionary note worth mentioning.^6^ In a related vein, Monitoring the Future students attended 360 public and private schools in rural *and* urban areas. As best I can tell, the ensuing analyses compared an essentially rural sample of American Indian students with their general population counterparts from rural and urban communities, with unknown implications for the conclusions.

Swaim and Stanley encourage various applications of this knowledge, primarily screening initiatives in health care settings. Indeed, the integration of behavioral health services and primary care has been promoted by private, state, and federal sponsors for more than 20 years. Of particular relevance is the Substance Abuse and Mental Health Services Administration’s Screening, Brief Intervention and Referral to Treatment effort, which has enabled large numbers of tribal health organizations to incorporate systematic procedures for detecting and managing substance use and abuse among American Indian youths and young adults.^7^ Local evaluations demonstrate successes within these settings but struggle to document broader, population-based impacts. It may be productive to determine whether schools that participate in the OYOF survey are located in communities that also support Screening, Brief Intervention and Referral to Treatment–like procedures in their clinics. The former could then potentially shed light on the wider impact of this novel intervention.

The questions of interest addressed by Swaim and Stanley represent a subset of those asked in the OYOF survey; presumably, other items inquire about risk and protective factors related to substance use among American Indian adolescents. Although beyond the scope of this article, I hope future analyses will probe the dynamics underpinning the contribution of these factors to the reported patterns of substance use. It is particularly important, consistent with today’s discourse in Native communities and broadening scientific inquiry, to consider the potentially mediating and moderating influences of personal resilience, assets, and other strengths that represent important resources for coping with the stressful environments of rural reservation life. Then, too, this work needs to move beyond egocentric attributes of individual youths to consider peer and family relationships, school climate and culture, institutional bonding, social connectedness, and collective competence as equally critical intervening variables.^8^ This emphasis is consistent with the growing recognition of the role of social determinants of health in exposure, vulnerability, resistance, onset, course, severity, consequences, and recovery.^9^ Doing so promises to yield further insights that can inform points of emphasis in prevention and early intervention.

The OYOF survey and the study by Swaim and Stanley expand the understanding of the dangers as well as promises that lie ahead for American Indian adolescents. The results offer not only a yardstick by which to measure our progress in improving the health and well-being of American Indian youths but also a roadmap by which to chart this journey for generations come.
